# Prevalence and Penetrance of *BRCA1* and *BRCA2* Germline Mutations in Colombian Breast Cancer Patients

**DOI:** 10.1038/s41598-017-05056-y

**Published:** 2017-07-05

**Authors:** D. Torres, J. Lorenzo Bermejo, M. U. Rashid, I. Briceño, F. Gil, A. Beltran, V. Ariza, U. Hamann

**Affiliations:** 1 Molecular Genetics of Breast Cancer, German Cancer Research Center (DKFZ), Heidelberg, Germany; 2 Institute of Human Genetics, Pontificia Universidad Javeriana, Bogota, Colombia; 3 Institute of Medical Biometry and Informatics, University of Heidelberg, Heidelberg, Germany; 4 Department of Basic Sciences Research, Shaukat Khanum Memorial Cancer Hospital and Research Centre (SKMCH & RC), Lahore, Pakistan; 5 Universidad de la Sabana, Bogota, Colombia; 6 Unit of Clinical Epidemiology and Biostatistics, Pontificia Universidad Javeriana, Bogota, Colombia; 7 Universidad Nacional, Bogota, Colombia

## Abstract

Pathogenic *BRCA1/2* germline mutations confer high risks of breast and ovarian cancer to women of European ancestry. Characterization of *BRCA1/2* mutations in other ethnic groups is also medically important. We comprehensively screened 68 Colombian breast/ovarian cancer families for small-range mutations, 221 families for large-genomic rearrangements, and 1,022 unselected breast cancer cases for Colombian founder mutations in *BRCA1/2*. The risk of cancer among relatives of mutation carriers and the mutation penetrance were estimated by survival analysis. Identified *BRCA2* mutations included 6310delGA and the recurrent 1991del4 mutations. A novel large *BRCA2* deletion was found in 0.9% of the screened families. Among unselected breast cancer cases, 3.3% tested positive for *BRCA1*/3450del4, 2.2% for *BRCA1*/A1708E, 1.1% for *BRCA2*/3034del4, and 0.4% for *BRCA2*/1991del4. Female relatives of carriers of *BRCA1/2* founder mutations showed a 5.90 times higher risk of breast cancer, when the woman herself carried a *BRCA1* mutation compared to a non-carrier (95% CI 2.01–17.3). The estimated cumulative risk of breast cancer by age 70 years for *BRCA1* mutations carriers was 14% (95% CI 5–38) compared to 3% for the general Colombian population (relative risk of breast cancer 4.05). Together with known founder mutations, reported novel variants may ease a cost-effective *BRCA1/2* screening in women with Colombian ancestry.

## Introduction

About 10% of breast cancers are hereditary and can be attributed to germline mutations in breast cancer susceptibility genes, in particular *BRCA1* and *BRCA2* (*BRCA1/2*). Accurate estimates of the risk of breast and ovarian cancers for *BRCA1/2* mutation carriers are crucial for genetic counselling. Preventive measures can be offered to women at high risk, such as intensified surveillance, prophylactic mastectomy and oophorectomy, and in some cases chemoprevention^1^. Several studies have investigated the risk of breast cancer in *BRCA1/2* mutation carriers. However, penetrances are usually estimated for mixtures of mutations found in women of European ancestry. Estimated cumulative risks for European women vary between 45% and 85% for breast cancer, and 10% and 65% for ovarian cancer by age 70 years, depending on the type of population and the type of case ascertainment^2–8^. Examination of founder mutations in non-European ethnic groups permits to assess mutation-specific penetrance, and possible differences between them, which may be of clinical relevance.

In Colombia, breast cancer is the main cause of cancer-related death among women, with incidence and mortality age-standardised (world) rates of 35.7 and 10.8 cases per 100,000 person-years, respectively^9^. The genome of Colombian women is the result of genetic admixture between Native Americans, Spaniards who reached South America in the sixteenth century, Native African slaves who arrived in seventeenth century, and subsequent immigration, mainly from Europe^10^. Little is known about the contribution of *BRCA1/2* mutations to hereditary breast cancer in Colombia. In a study on 53 breast/ovarian cancer families, we previously identified three common deleterious founder mutations, 3450del4 and A1708E in *BRCA1*, and 3034del4 in *BRCA2* (BIC nomenclature (https://research.nhgri.nih.gov/bic/)^11^. The two founder *BRCA1* mutations accounted for 100% of all *BRCA1* mutations, and the identified founder *BRCA2* mutation represented 40% of all *BRCA2* mutations in this initial set of families. The overall prevalence of *BRCA1/2* mutations was 50% in multiple case breast cancer families and 33% in breast and ovarian cancer families. Two studies of unselected breast (and ovarian) cancer patients reported *BRCA1/2* mutation frequencies of 1.2% and 15%, respectively^12, 13^. In these studies, the two Colombian *BRCA1* founder mutations accounted for 100% (breast cancer patients) and 92% (ovarian cancer patients) of all *BRCA1* mutations.

The lack of data on possible large-genomic rearrangements (LGRs) in *BRCA1/2*, and the scarcity of data on the prevalence of *BRCA1/2* mutations in Colombian familial and unselected breast cancer patients motivated the present study. We comprehensively screened 68 breast/ovarian cancer families for small-range mutations, 221 families for LGRs, and 1,022 unselected breast cancer cases for Colombian founder mutations in *BRCA1/2*. We conducted survival analyses to estimate the risk of cancer among relatives of carriers of *BRCA1/2* mutations, and the penetrance of specific Colombian *BRCA1/2* founder mutations.

## Results

### Spectra and Frequencies of *BRCA1/2* Mutations in Breast/Ovarian Cancer Families

In total, 290 index cases from 289 breast/ovarian cancer families were investigated. Table 1 shows their high-risk group distribution. Twelve patients were diagnosed before 35 years of age; 242 belonged to families with at least two breast cancer cases; 34 patients to families with both, breast and ovarian cancer; one to a family with male breast cancer; and one to a family with at least one ovarian cancer. The median age of disease onset was 46 years (range 25–83) for female breast cancer (n = 288). The male patient was diagnosed with breast cancer at 69 years of age and the ovarian cancer patient at 53 years of age.

**Table 1:** Distribution of examined families in high-risk groups and corresponding *BRCA1/2* mutation frequencies.

Risk Group	Family Phenotype	Screened for Small-Range Mutations				Screened for Large-Genomic Rearrangements			
		Number of families	Number (%) with mutations in			Number of Families	Number (%) with mutations in		
			*BRCA1*	*BRCA2*	*BRCA1/2*		BRCA1^*a*^	*BRCA2*	*BRCA1/2*
	**Female BC families**	**57**	0 (0.0)	6 (10.5)	6 (10.5)	**196**	0 (0.0)	**2** (**1**.**0**)	**2** (**1**.**0**)
A1	1 case ≤ 35 years	0	0 (0.0)	0 (0.0)	0 (0.0)	12	0 (0.0)	0 (0.0)	0 (0.0)
A2	Multiple cases	57	0 (0.0)	6 (10.5)	6 (10.5)	184	0 (0.0)	2 (1.0)	2 (1.0)
A3	**Breast**-**ovarian cancer families**	**10**	1 (10.0)	0 (0.0)	1 (10.0)	**24**	0 (0.0)	0 (0.0)	0 (0.0)
	≥1 BC and ≥ 1 OC, at any age								
B	**Male BC families**	0	0 (0.0)	0 (0.0)	0 (0.0)	**1**	0 (0.0)	0 (0.0)	0 (0.0)
	≥1 case of male BC								
C	**OC families**	**1**	0 (0.0)	0 (0.0)	0 (0.0)	0	0 (0.0)	0 (0.0)	0 (0.0)
	≥1 OC at any age								
	**All families**	**68**	1 (1.5)	6 (8.8)	7 (10.3)	**221**	0	2 (0.9)	2 (0.9)

BC, breast cancer; OC, ovarian cancer.

^a^72/221 breast/ovarian cancer families were screened for *BRCA1*.

Screening of the complete *BRCA1/2* coding regions in 68 families revealed seven (10%) deleterious mutations: one in *BRCA1* and six in *BRCA2*. Risk-group specific *BRCA1/2* mutation frequencies are shown in Table 1. The recurrent *BRCA2/*1991del4 mutation accounted for 33% of all *BRCA2* mutations identified in this family set (Table 2). All mutations were frame shift mutations. The *BRCA2* 6310delGA mutation has been previously reported once in the NCBI mutation database. Similar ages at diagnosis were found in *BRCA1/2* mutation carriers (n = 8, median 53 years, range 38–65 years) and non-*BRCA* carriers (n = 60, median 56 years, range 32–83 years).

**Table 2:** Small-range mutations and large genomic rearrangements in the *BRCA1/2* genes in Colombian breast/ovarian cancer families.

Family	Gene	Mutation Nomenclature			Classification^c^	No. of BIC Entries^b^	
		BIC^a^: genomic level	HGVS^b^: genomic level	HGVS^b^: protein level		Total^d^	with Hispanic ancestry^e^
**Deleterious small**-**range mutations**							
295	*BRCA1*	1793delA	c.1674delA	p.Gly559fs	M	6	5
291,409	*BRCA2*	1991del4	c.1763_1766delATAA	p.Asn588fs	M	5	1
382	*BRCA2*	2929delC	c.2701delC	p.Ala902fs	M	^f^	1
55/648^g^	*BRCA2*	6252insG	c.6024dupG	p.Gln2009fs	M	2	2
399	*BRCA2*	6306delAA	c.6078_6079delAA	p.Glu2028fs	M	3	1
282	*BRCA2*	6310delGA	c.6082_6083delGA	p.Glu2028fs	M	2	1^j^
**Sequence variants of no or uncertain clinical significance**							
644	*BRCA2*	451G > C	c.223G > C	p.Ala75Pro	benign	52	8
643	*BRCA2*	IVS4 − 37T > A	c.426 − 37T > A	—	VUS	2	1
23,639	*BRCA2*	IVS13 − 62A > G	c.7008 − 62A > G	—	benign/VUS	6	1
237	*BRCA2*	IVS14 + 53C > T	c.7435 + 53C > T	—	benign	39	1
430	*BRCA2*	IVS19+15T> C	c.8487+15T> C	—	benign^h^	Novel	1^l^
558	*BRCA2*	IVS21 − 19A > G	c.8755 − 19A > G	—	VUS	^i^	1
31,92,482,545	*BRCA2*	IVS24 − 83G > A	c.9257 − 83 G > A	—	benign	3	1
485	*BRCA2*	IVS24 − 143T > A	c.9257 − 143T > A	—	benign	^j^	1
**Large genomic rearrangements**							
0055,1465	*BRCA2/*ex1-14del^k^				M	Novel	1^l^

^a^BIC, Breast Cancer Information Core database as of October 2016 (https://research.nhgri.nih.gov/bic/).

^b^Nomenclature follows Human Genome Variation Society (HGVS) (https://www.hgvs.org/). Numbering starts at the first A of the first coding ATG. (located in exon 2) of NCBI GenBank Accession NM_007294.3 (*BRCA1*) and NM_000059.3 (*BRCA2*).

^c^M, deleterious mutation; VUS, variant of uncertain clinical significance.

^d^Including those with ancestry data and those from the present study.

^e^The term “Hispanic” was used for individuals of Spanish, Mexican, Central and South American, Cuban, or Puerto Rican descent.

^f^Reported in one multiple case breast cancer family from Spain (in Miramar MD, *et al*. Genetic analysis of BRCA1 and BRCA2 in breast/ovarian cancer families. from Aragon (Spain). Breast Cancer Res Treat 2008;112(2):353-8, p353).

^g^Two probands in family 55/648.

^h^Classification based on *in silico* analyses.

^i^Three times reported in NCBI.

^j^Once reported in NCBI.

^k^The deletion breakpoints were not determined.

^l^Identified in the present study.

In addition to the deleterious mutations, eight distinct *BRCA2* sequence variants were identified. Seven variants had already been detected in recent studies and classified as benign or as variants of uncertain clinical significance (VUS). The novel variant, IVS19+15T > C, was predicted to be benign by 5/5 *in silico* prediction tools.

Multiplex ligation dependent probe amplification (MLPA) screening for LGRs in the *BRCA1/2* genes was performed in index cases of 221 breast/ovarian cancer families. Table 1 shows their high-risk group distribution. One novel large *BRCA2* deletion, comprising exons 1 to 14 (ex1-14del), was identified in two (0.9%) unrelated patients (Supplementary Figure 1). No LGRs were identified in *BRCA1*. Phenotypes of all families harbouring deleterious *BRCA1/2* germline mutations are shown in Table 3.

**Table 3:** Characteristics of the Colombian breast/ovarian cancer families harboring *BRCA1/2* mutations and variants.

Family	No. of Cancers		Age at Onset (years)		Other Cancers: Age at Onset (years)
	Female BC (bilateral)	OC	BC	OC	
***Families carrying deleterious BRCA1 mutations***					
295	5	1	38*, 61, ?, ?, ?	39	Colon:65, prostate:83
***Families carrying deleterious BRCA2 mutations***					
*Small*-*range mutations*					
291	3	—	30, 35, 65*	—	Sarcoma:47
399	3	—	44*, 60, 74	—	—
409	3	—	43, 45*, 45	—	3x Skin:48, 50, 89, colon:33, lung:69
382	3 (1)	—	47, 55*, 55/65	—	Leukemia:55
55^a^/648	4	—	46*, 63*, 82, ?	—	Colon:31, 2x cervix:40, ?, stomach:41, esophagus:83
282	4 (1)	—	34/65*, 60, 60, 68	—	Colon:30, bladder:65, lung:73
*Ex1*-*14del mutations*					
1465	3	—	47, 51*, 61	—	Liver:60, retinoblastoma:?
0055	3 (1)	—	45/48*, 48, 64	—	—
***Families carrying BRCA2 sequence variants***					
482	2	1	55, ?	59*	—
31	3	—	36, 38, 47*	—	Larynx:40
92	3	—	45, 50*, 70	—	Larynx:75
430	3	1	38, 40, 45*	79	—
545	3	—	35*, 37, ?	—	Lung:?
558	3	—	58, 62, 68*	—	—
644	3	—	55, 60, 63*	—	Liver:63, 2x colon:66, 70, pancreas:70
643	3	—	54, 55*, 55	—	Thyroid:54
237	4	—	48, 63*, 66, 68	—	—
23	5	—	56, 57*, 58, 63, 75	—	Brain:63, bone:79, larynx:80, prostate:86
485	6	—	37, 40, 49*, ?, ?, ?	—	Lung:?, liver:?
639	6	—	40, 40, 40, 49, 50*, 50	—	—

^*^Proband, BC: breast cancer, OC: ovarian cancer.

^a^Case 648 from the Col-BCCC study turned out to be a member of family 55.

**Figure 1:**
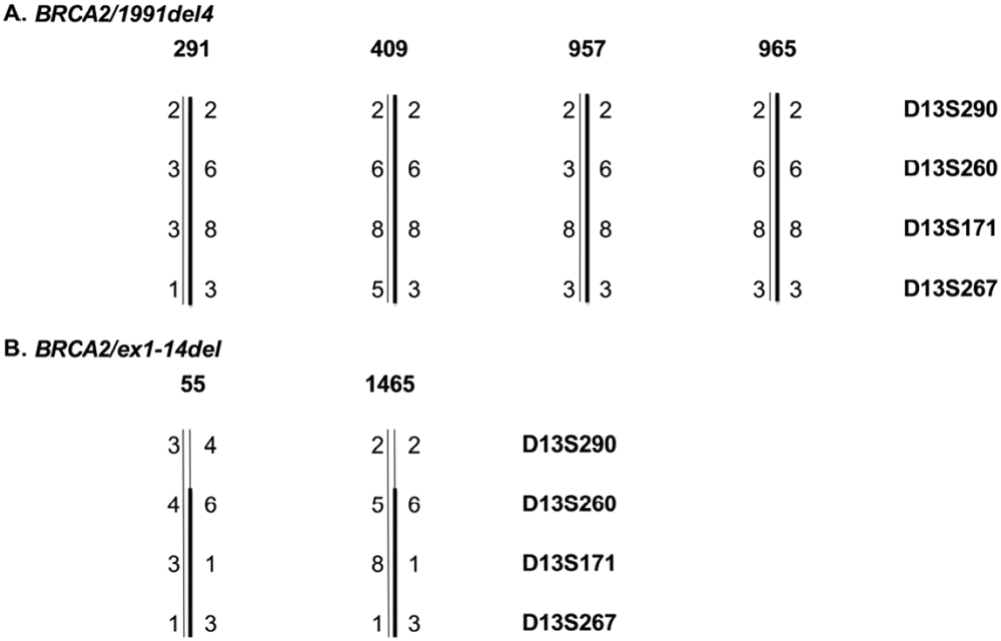
Haplotype analysis of *BRCA2/*1991del4 (**A**) and *BRCA2/*ex1-14del (**B**) on mutation carriers at four microsatellite *BRCA2* flanking loci. Family numbers are given above the haplotype. Alleles are coded by numbers. D13S290: allele 2 (CA)_12_, allele 3 (CA)_13_, allele 4 (CA)_14_; D13S260: allele 3 (CA)_20_, allele 4 (CA)_21_, allele 5 (CA)_22_, allele 6: (CA)_23_; D13S171:allele: 1 (CA)_13_, allele 3 (CA)_15_, allele 8 (CA)_20_; D13S267: allele 1 (CA)_32_, allele 3 (CA)_34_, allele 5 (CA)_36_. Common haplotypes are indicated by a bold bar.

Haplotype analysis for the two recurrent *BRCA2* mutations, 1991del4 and ex1-14del was performed on all mutation carriers at four *BRCA2* flanking loci. *BRCA2*/1991del4 was identified in four breast cancer patients and ex1-14del in two breast cancer patients. All four 1991del4 mutation carriers shared the same haplotype. The two ex1-14del mutation carriers displayed a similar allelic pattern at three loci and a distinct pattern at one locus (Fig. 1).

### Spectra and Frequencies of *BRCA1/2* Mutations in Unselected Breast Cancer Patients

The frequency of the four small-range *BRCA1/2* Colombian founder mutations was assessed in 1,022 unselected breast cancer patients participating in the Colombian breast cancer case-control study (Col-BCCC) using PCR-based methods. In total, 71 (7%) *BRCA1/2* mutations were identified: 56 (5.5%) in *BRCA1* and 15 (1.5%) in *BRCA2*. Mutation frequencies are shown in Table 4. No mutations were detected in the 1,023 healthy Col-BCCC controls.

**Table 4:** Frequencies of the four small-range *BRCA1/2* founder mutations in unselected breast cancer patients and controls from Col-BCCC.

Gene	Mutation Nomenclature			No. of Mutations (%) in Cases (n = 1,022)	No. of Mutations (%) in Controls (n = 1,023)
	BIC^a^: genomic level	HGVS^b^: genomic level	HGVS^b^: protein level		
*BRCA1*	3450del4	c.3331_3334delCAAG	p.Gln1111fs	34 (3.3)	0 (0)
*BRCA1*	A1708E	c.5123C > A	p.Ala1708Glu	22 (2.2)	0 (0)
*BRCA2*	1991del4	c.1763_1766delATAA	p.Asn588fs	4 (0.4)	0 (0)
*BRCA2*	3034del4	c.2808_2811delACAA	p.Ala938fs	11 (1.1)	0 (0)
Total no. of mutations				**71** (**7**.**0**)	**0** (**0**)

^a^BIC, Breast Cancer Information Core database as of October 2016 (https://research.nhgri.nih.gov/bic/).

^b^HGVS, Human Genome Variation Society (https://www.hgvs.org/).

### Penetrance of Colombian *BRCA1/2* Founder Mutations

The risk of breast cancer, ovarian cancer, and any type of cancer, including cervical cancer and melanoma, was first assessed in 251 female relatives of 73 carriers of founder mutations in *BRCA1/2* (probands). Seventeen relatives of *BRCA1/2* mutation carriers were diagnosed with primary breast cancer, four with primary ovarian cancer, two with melanoma and three with cervical neoplasms. Table 5 shows estimated hazard ratios (HR) of breast cancer from a multiple Cox proportional hazards model that in addition to the presence and type of *BRCA1/2* mutations included the covariates birth year, type of recruitment (family or unselected case-control study), type of relationship with the proband as well as proband’s age at diagnosis. A minority of the relatives of *BRCA1/2* mutation carriers (n = 38) was enrolled within the Col-BC/OC-Family study; the majority (n = 213) were recruited through unselected probands from the Col-BCCC study. The investigated cohort included eleven mothers, 86 sisters and 57 daughters (first-degree relatives) as well as 97 second- and third-degree relatives (aunts, nieces, grand-mothers, grand-daughters) of mutation carriers.

**Table 5:** Estimated hazard ratios (HRs) of breast cancer in relatives of carriers of Colombian *BRCA1/2* founder mutations stratified by birth year, proband’s age at diagnosis, mutated *BRCA1/2* gene and *BRCA1/2* mutation type.

Variable	Level	Women	Events	HR	95% CI	Pval	Cumulative Risk by Age 70 Years	95% CI
Birth year	Before 1960	65	6	Ref.		0.17	0.21	0.00–0.39
	1960–69	59	10	3.94	1.19–13.1		0.60	0.00–0.90
	1970–79	53	1	2.67	0.25–28.3		0.47	0.00–0.89
	1980+	74	0	—				
Study type	Case-control	213	13	Ref.		0.74	0.25	0.02–0.42
	Family study	38	4	1.22	0.39–3.79		0.29	0.00–0.52
Relationship with proband	Other	97	7	2.72	0.91–8.13	0.07	0.52	0.00–0.78
	Sister	86	6	Ref.			0.23	0.00–0.44
	Daughter	57	4	5.53	1.51–20.2		0.77	0.00–0.97
	Mother	11	0	—				
Proband’s age at diagnosis	Less than 40	59	3	Ref.		0.37	0.21	0.00–0.42
	40–44	57	4	1.01	0.23–4.52		0.21	0.00–0.41
	45–49	70	3	1.09	0.22–5.44		0.23	0.00–0.46
	50+	65	7	2.47	0.63–9.61		0.44	0.00–0.71
*BRCA1/2* mutation	None	142	5	Ref.		0.01	0.13	0.00–0.25
	*BRCA1*	80	10	5.90	2.01–17.3		0.55	0.04–0.79
	*BRCA2*	29	2	2.55	0.49–13.1		0.30	0.00–0.59
Mutation type	None	142	5	Ref.		0.02	0.13	0.00–0.26
	3450del4	54	7	5.63	1.78–17.8		0.55	0.00–0.80
	A1708E	26	3	6.66	1.57–28.4		0.61	0.00–0.90
	1991del4	7	1	8.45	0.97–73.2		0.70	0.00–0.98
	3034del4	22	1	1.50	0.17–12.8		0.19	0.00–0.48

HR, hazard ratio; CI, confidence interval; Pval, P-value; Ref., reference.

In total, 109 relatives of mutation carriers were found to carry themselves *BRCA1/2* mutations (80 in *BRCA1* and 29 in *BRCA2*). Among first-degree relatives of mutation carriers, 44 carried *BRCA1* and 18 *BRCA2* mutations. Statistically significant risk differences were found between *BRCA1/2* mutation carriers and non-carriers. The HR of breast cancer was 5.90 (95% CI 2.01 to 17.33) for *BRCA1* and HR = 2.55 (95% CI 0.49 to 13.13) for *BRCA2* mutation carriers compared to non-carriers. Risk differences were also noticed when relatives of probands were stratified by mutation type. The highest risk of breast cancer was found in carriers of the *BRCA2/*1991del4 mutation (HR = 8.45, 95% CI 0.97 to 73.24) followed by the two *BRCA1* mutations A1708E (HR = 6.66, 95% CI 1.57 to 28.38) and 3450del4 (HR = 5.63, 95% CI 1.78 to 17.81), and the *BRCA2*/3034del4 mutation (HR = 1.50, 95% CI 0.17 to 12.84).

Separate analyses were performed to evaluate the cumulative risk of ovarian cancer by age 45 years in first-degree relatives of *BRCA1/2* mutation carriers. It amounted to 5% for sisters (three ovarian cancer diagnoses) and 12% for daughters (one ovarian cancer diagnosis) of probands. Complete results for ovarian cancer and combined cancer types (breast, ovarian and cervical cancers, and melanoma) are provided in Supplementary Tables 1 and 2.

Penetrance analyses of the complete set of pedigrees using Mendel resulted in a HR of breast cancer by age 70 years equal to 2.81 (95% CI 1.47 to 5.35) for carriers of founder *BRCA1* mutations compared to non-carriers. Together with a cumulative breast cancer risk of 3.3% in the general Colombian population, this translates into a 9% penetrance by age 70 years (95% CI 5 to 18). Visual inspection of pedigree deviances revealed four departing pedigrees, two of them in favour of the alternative (with two carriers each diagnosed at ages 32 and 41, and 29 and 38 years), and two of them in favour of the null hypotheses (with a non-carrier diagnosed at age 39 and one unaffected carrier at age 60 years) (Supplementary Figure 2). Exclusion of these four outlying families increased the HR of breast cancer by age 70 years to 4.05 (95% CI 1.43 to 11.4), and the corresponding cumulative risk of breast cancer by age 70 years to 14% (95% CI 5 to 38). The number of non-proband women diagnosed with breast cancer was too small to estimate the penetrance of *BRCA2* mutations (two non-proband cases), and separate *BRCA1* mutations (n = 7 for 3450del4 and n = 3 for A1708E).

## Discussion

To our knowledge, this is the largest study on the prevalence of *BRCA1/2* mutations in breast/ovarian cancer families and unselected breast cancer patients from Colombia. It is also the first report on the prevalence of LGRs in *BRCA1/2*, and on the cancer risk conferred by specific Colombian *BRCA1/2* founder mutations. In the present study, seven deleterious small-range *BRCA1/2* mutations were identified in 10% of Colombian breast/ovarian cancer families, including the recurrent *BRCA2/*1991del4 mutation, which showed a founder origin. This mutation has been identified previously in a single African American multiple case breast cancer family and more recently in a single breast and ovarian cancer family from Serbia^14, 15^. A novel LGR *BRCA2*/ex1-14del was found in 0.9% of Colombian breast/ovarian cancer families. A higher LGR frequency of 6.7% was recently reported in a large study on 1,560 Latin American/Caribbean breast/ovarian cancer families, which may be due to different selection criteria, since only families with at least three breast/ovarian cancer cases were screened in the present study, and to ethnic differences^16^.

Identified *BRCA2*/ex1-14del mutation carriers shared a conserved haplotype over an approximately 4 cM region spanning the *BRCA2* locus implying that the mutation may have arisen from a common founder. *BRCA2*/ex1-14del, identified in Colombian breast/ovarian cancer families, represents the second founder LGR identified in a Hispanic population after *BRCA1/*ex9-12del, which is a common Mexican founder mutation^17, 18^.

Interestingly, none of the LGRs previously identified in Hispanic breast/ovarian cancer families and unselected breast cancer patients have been found in Colombians^19–21^. Previously identified LGRs include *BRCA1/*ex10dup and amplification of *BRCA1* exons 3, 5 and 6 in Chileans^22^, *BRCA1/*ex9-12del in Mexican Americans^18^, *BRCA1*/ex8-9dup^17^, *BRCA1*/ex18-19del, and *BRCA1*/ex8-10del in Mexicans^17^, *BRCA2/*ex1-2del in Costa Ricans^23^, *BRCA1*/ex8-9del found in Bahamians^24^ and *BRCA2/*ex14del detected in male breast cancer patients from Brazil^25^. In short, it seems that the spectrum of LGRs varies among Latin Americans and among Latino Americans.

Combination of previously reported and present prevalences of small-range *BRCA1/2* founder mutations result in 89% of all *BRCA1* mutations attributed to A1708E and 3450del4, and 44% of all *BRCA2* mutations due to the novel 1991del4 and 3034del4 mutations^11^. Inclusion of the two novel founder mutations *BRCA2/*1991del4 and *BRCA2*/ex1-14del in routine *BRCA1/2* mutation testing would improve risk assessment and carrier detection in Colombian women.

The four most common *BRCA1* mutations in Latin American breast cancer patients are: the ex9-12del mutation (1.45%) found in Mexican Americans and Mexicans, but not in Spaniards and South Americans^19, 21, 26^; 185delAG (0.9%) is found in many different regions of Latin America including Argentina, Brazil, Chile, Mexico and Peru; A1708E (0.58%) is found in Mexico, Spain and Colombia (one of the Colombian founder mutations)^11, 18, 27^; 3450del4 (0.15%), finally, is identified in patients from Brazil, Chile and Colombia (one of the Colombian founder mutations). For *BRCA2*, the most common mutations are H372N (0.88%), E49X (0.38%), 3492instT (0.37%), and 6174delT (0.32%). The H372N and 6174delT mutations have been found in Argentina, Brazil, Chile, Costa Rica, but not in Mexico, while 3492insT was found in Mexico and not in any other Latin American country^23, 28^. The 3034del4 (0.07%) was identified in Argentina, Colombia (one of the Colombian founder mutations) and Peru. These results demonstrate that certain mutations are specific of certain regions, whereas others are found throughout the whole Latin America. However, in consideration that organized genetic *BRCA1/2* testing is not performed in most Latin American countries, further studies are required to investigate regional differences.

In the present study, the frequencies of the Colombian founder mutations (*BRCA1/*A1708E, *BRCA1/*3450del4, *BRCA2/*1991del4, *BRCA2*/3034del4) in 1,022 unselected breast cancer cases were 5.5% for *BRCA1* and 1.5% for *BRCA2*. No mutations were identified in 1,023 controls. The cumulative frequency of the two *BRCA1* founder mutations is likely slightly smaller than the frequency that would have been obtained in a complete gene screen, given that the two *BRCA1* mutations account for approximately 90% of all *BRCA1* mutations. In contrast, the 1.5% *BRCA2* mutation frequency is likely underestimated, as the two screened *BRCA2* mutations only account for about half of all *BRCA2* mutations. Other studies on the prevalence of *BRCA1/2* mutations in unselected breast cancer patients from Brazil (n = 402), Mexico (n = 810; n = 96), Colombia (Medellin) (n = 244), Cuba (n = 307) and Peru (n = 266) have reported frequencies in the range 0.3–11.4% for *BRCA1* and 0.4–3.1% for *BRCA2* mutations^12, 13, 17, 29–32^. Probably also these frequencies have been underestimated, given that *BRCA1/2* genes were only partially screened, few mutations or panels of known mutations were tested, and screening was restricted to few LGRs or was not performed. A remarkably higher frequency of *BRCA1* mutations equal to 24% has been reported in the Bahamas^24, 33^.

We found that the highest risk of breast cancer was associated with the frame shift mutation *BRCA2/*1991del4, but this result needs validation due to the small number of women investigated in the analysis. Both the estimated relative risk of breast cancer by age 70 years (4.05) and the corresponding cumulative risk for *BRCA1* mutation carriers (14%) were lower than expected. Previously reported cumulative risks of breast cancer for *BRCA1* mutation carriers vary largely between studies, with an average cumulative risk of 65%^2^. Possible reasons for the risk differences with earlier reports include the study design, applied methodology and the investigated Colombian population, which carries specific types of *BRCA1* mutations and possibly particular genetic modifiers^2–8^. Risk differences could be also related to specific environmental modifiers, including the larger number of children, younger age at first birth, shorter height, less hormone use, and less alcohol consumption of Hispanic women compared to non-Hispanic white women^34^. External validation on an independent set of mutation-positive families is needed before interpreting screening implications.

Breast cancer is the most common cancer worldwide, and Latin America is not an exception, with rising incidence and mortality rates. Today, a commercial screening test for three previously identified founder small-range mutations is used. We propose to extend this panel by incorporating the novel founder mutations identified in this study (1991del4 and ex1-14del in *BRCA2*). These novel mutations should be also included in the panel of 114 recurrent Hispanic *BRCA1/2* mutations (HISPANEL). A less-expensive *BRCA1/2* testing tool would constitute a cost-effective strategy to further enhance control of breast and ovarian cancer in women with Colombian ancestry.

## Methods

### Ethical Approval

Informed consent was signed by all study participants. The research protocol was approved by the Ethics Committee of the Pontificia Universidad Javeriana in Bogota, Colombia. The methods were carried out in accordance with relevant guidelines and regulations.

## Patients and Methods

### Study Populations

Breast/ovarian cancer families were ascertained within a study (Col-BC/OC-Family) at the Institute of Human Genetics, Pontificia Universidad Javeriana, Bogota, Colombia from June 2004 to January 2008. Eighty-four index patients from 83 breast and/or ovarian cancer families, diagnosed with invasive breast or epithelial ovarian cancer, were selected for genetic testing after genetic counselling. The patient collective included 41 newly recruited families, and 42 index cases from 42 families, who previously tested negative for small-range *BRCA1/2* mutations^11^.

Unselected patients were recruited within the Col-BCCC, which included 1,022 breast cancer cases and 1,023 controls. Cases were mainly recruited from hospitals in Bogota, Neiva and Villavicencio, which are located in the geographic centre of Colombia (the so called Andean region), during the period 03/2007 to 02/2011. Cases were women with a diagnosis of breast cancer after January 1^st^, 2004. Controls were recruited in the period of 06/2007 to 06/2011. Controls were healthy and unrelated women, who reported no family history of breast or any other type of cancer in two generations and who participated in the Colombian National Pap Smear Program^35^. Cases and controls were eligible if they were of Hispanic origin and resided in the study region. Controls were matched to cases by 2-year age classes.

### DNA Isolation and *BRCA1/2* Mutation Analyses

Genomic DNA was extracted from nine millilitre of peripheral blood collected into an EDTA tube using the salting out extraction method^36^. The entire coding regions and adjacent intronic splice junctions of *BRCA1* (Genbank accession number U14680) and *BRCA2* (Genbank accession number U43746) were screened in 69 index patients from 68 breast/ovarian cancer families (41 newly recruited within the Col-BC/OC-Family study, and 27 from the Col-BCCC study in A2, A3, C high-risk families). We used denaturating high performance liquid chromatography (DHPLC) analysis with the WAVE DNA fragment analysis system (Transgenomics, Omaha, NE, USA). PCR-primer pairs, PCR reactions set-up, cycling conditions, and DHPLC running conditions were as previously described^37, 38^. Each sample revealing variants was sequenced using an automated DNA CEQ 8000 sequencer (Beckman, Hilden, Germany) as previously described^11^.

Two hundred and twenty-one index patients from breast/ovarian cancer families (42 from the Col-BC/OC-Family study, who tested negative for small-range *BRCA1/2* mutations, and 179 from the Col-BCCC study, who belonged to A1-A3, B high-risk families) were further screened: 72 for LGRs in *BRCA1* and 221 for LGRs in *BRCA2*. MLPA analysis was performed using probe mix P002 and P087 for *BRCA1* and P045 for *BRCA2* according to the manufacturer’s instructions (MRC Holland, Amsterdam, the Netherlands). PCR was carried out using D4-labeled primers. Separation and relative quantification of the amplified product was obtained using the Beckman CEQ 8000XL DNA Analysis System (Beckman Coulter, Fullerton, USA). Variation in peak height was evaluated by comparing each sample with normal controls using SeqPilot software (JSI medical systems, Kippenheim, Germany). The presence of a deletion was confirmed by a second MLPA.

The prevalence of the four Colombian founder mutations was assessed in 1,022 unselected breast cancer cases and 1,023 healthy controls of the Col-BCCC study. Screening for *BRCA1/*3450del4, *BRCA2/*1991del4 (identified in present study), and *BRCA2*/3034del4 was performed by mismatch PCR. Screening for *BRCA1/*A1708E was performed by PCR-based restriction fragment length polymorphism (RFLP) analysis.

For *BRCA1/*3450del4, the PCR reaction was set up in 10 µl containing 25 ng genomic DNA, 1x PCR buffer (Green GoTaq Flexi Buffer, Promega, Madison, WI, USA), 1.5 mM MgCl_2_, 250 µM of dNTPs, 0.1 µM of each allele specific primer, 0.3 µM of the mismatch primer and 1 U DNA Polymerase (GoTaq Hot Start Polymerase, Promega). After an initial GoTaq activation step for 10 minutes at 95 °C, 30 cycles of PCR reactions consisting of 1 minute at 94 °C, 1 minute at 55 °C and 1 minute at 72 °C were carried out. This was followed by a final extension step of 7 minutes at 72 °C. Amplified PCR products were separated on a 3% agarose gel containing ethidium bromide and scored by UV visualisation. The fragment sizes of the wild type and mutant alleles were 287 bp and 162 bp, respectively

PCR reactions and conditions for *BRCA2/*1991del4 and *BRCA2*/3034del4 were as for *BRCA1/*3450del4 with the exception of using 2 mM and 3 mM MgCl_2_, respectively. The fragment sizes of the wild type and mutant alleles were 233 bp and 144 bp for *BRCA2/*1991del4 and 342 bp and 153 bp for *BRCA2*/3034del4.

PCR reactions and conditions for *BRCA1/*A1708E (C > A) were as for *BRCA1/*3450del4 with the exception of using two allele specific PCR primers, 3 mM MgCl_2_, and an annealing temperature of 60 °C. Amplified PCR products were digested with 15 U *Aci*I in a total volume of 22 µl (New England BioLabs, Ipswich, MA, USA) for 12 hours at 37 °C, followed by 20 minutes at 60 °C to inactivate *Aci*I and separated on a 3% agarose gel containing ethidium bromide. The fragment sizes of the wild type C allele were 226 bp and 126 bp and of the mutant A allele 352 bp.

Primer sequences are available upon request. All primers were synthesized by Integrated DNA Technologies, Coralville, IA, USA.

### Haplotype Analysis

Individuals with identical *BRCA2* germline mutations from apparently unrelated families were scored for allele sharing indicative of a common ancestor. Haplotype analysis was performed at four extragenic microsatellite loci D13S290, D13S260, D13S171, and D13S267) flanking the *BRCA2* gene. Microsatellite alleles were identified by automated fluorescent-bases fragment detection from amplified PCR products using a CEQ 8000 XL DNA Analysis System (Beckman, Hilden, Germany).

### *In silico* Analyses

The novel *BRCA2* variant in intron 19 was evaluated for its potential effect on splicing using the splice prediction algorithms SpliceSiteFinder-like (http://www.umd.be/-searchSplice-Site.html), MaxEntScan (http://genes.mit.edu/burgelab/maxent/), NNSPLICE (http://www.fruitfly.org/seq_tools/splice.html), GeneSplicer (http://ccb.jhu.-edu/software/genesplicer/), and HumanSplice Finder (http://www.umd.be/HSF/). We used the Alamut software interface (Interactive Biosoftware) with default settings.

### Statistical Analyses

Two different approaches were used to assess the risk of cancer conferred by Colombian *BRCA1/2* founder mutations. First, a standard multiple Cox proportional hazards model was fitted to censored information on age at diagnosis from relatives of carriers of *BRCA1/2* founder mutations (probands), and cumulative risks were estimated using Breslow method. We also estimated the penetrance of *BRCA1* mutations with the Mendel package for statistical analyses, taking into account ascertainment by conditioning on proband diagnoses^39, 40^. In short, a Cox proportional hazards model was fitted to censored data on age at diagnosis from probands and their relatives, incorporating the background incidence of breast cancer in Colombia reported by GLOBOCAN as the baseline hazard function^9^. Mendel allows multiple probands per pedigree and conditions on specially appended pedigrees during parameter estimation in a modified segregation analyses. We visually inspected the deviance (twice the log-likelihood difference under the alternative and null hypotheses) of each pedigree in order to identify influential families in favour or against the null hypothesis of no risk increase.

## Electronic supplementary material

Supplementary Material
