# Supplementary material for: Prevalence and Penetrance of BRCA1 and BRCA2 Germline Mutations in Colombian Breast Cancer Patients
Source: Sci Rep. 2017 Jul 5;7:4713. doi: 10.1038/s41598-017-05056-y (PMC5498630; doi:10.1038/s41598-017-05056-y)

Prevalence and Penetrance of *BRCA1* and *BRCA2* Germline Mutations in Colombian Breast Cancer Patients

D. Torres[1,2,§], J. Lorenzo Bermejo[3,§], M. U. Rashid[1,4], I. Briceño[2,5], F. Gil[6], A. Beltran[7], V. Ariza[7], U. Hamann[1]

[1] Molecular Genetics of Breast Cancer, German Cancer Research Center (DKFZ), Heidelberg, Germany

[2] Institute of Human Genetics, Pontificia Universidad Javeriana, Bogota, Colombia

[3] Institute of Medical Biometry and Informatics, University of Heidelberg, Heidelberg, Germany

[4] Department of Basic Sciences Research, Shaukat Khanum Memorial Cancer Hospital and Research Centre (SKMCH & RC), Lahore, Pakistan

[5] Universidad de la Sabana, Bogota, Colombia

[6] Unit of Clinical Epidemiology and Biostatistics, Pontificia Universidad Javeriana, Bogota, Colombia

[7] Universidad Nacional, Bogota, Colombia

§ These authors contributed equally to this work.

Correspondence**:** u.hamann@dkfz-heidelberg.de

Supplementary Data

Supplementary Table 1. Estimated hazard ratios (HRs) of ovarian cancer in relatives of carriers of Colombian *BRCA1/2* founder mutations.

Supplementary Table 2. Estimated hazard ratios (HRs) of breast, ovarian and cervical cancer, and in relatives of carriers of Colombian *BRCA1/2* founder mutations.

Supplementary Figure 1. Multiplex ligation-dependent probe amplification (MLPA) analysis of the *BRCA2* gene.

Supplementary Figure 2. Pedigree deviances.

Supplementary Table 1. Estimated hazard ratios (HRs) of ovarian cancer in relatives of carriers of Colombian *BRCA1/2* founder mutations stratified by birth year, proband's age at diagnosis, mutated *BRCA1/2* gene and *BRCA1/2* mutation type.

Variable	Level	Women	Events	HR	95%	CI	Pval	Σrisk45y	95%	CI
Birth year	Before 1960	65	1	Ref.			0.34	0.02	0.00-	0.04
	1960-69	59	3	5.60	0.57-	55.35		0.08	0.00-	0.17
	1970-79	53	0	-						
	1980+	74	0	-						
Study type	Case-control	213	3	Ref.			0.80	0.04	0.00-	0.08
	Family study	38	1	1.35	0.14-	12.98		0.05	0.00-	0.14
Relationship with proband	Other	97	0	-			0.87			
	Sister	86	3	Ref.				0.05	0.00-	0.10
	Daughter	57	1	2.64	0.27-	25.48		0.12	0.00-	0.32
	Mother	11	0	-						
Proband's age at diagnosis	Less than 40	59	0	-			0.90			
	40-44	57	1	Ref.				0.03	0.00-	0.09
	45-49	70	2	2.38	0.22-	26.22		0.08	0.00-	0.17
	50+	65	1	1.23	0.08-	19.72		0.04	0.00-	0.11
BRCA1/2 mutations	None	142	1	Ref.			0.17	0.01	0.00-	0.04
	BRCA1	80	3	8.94	0.93-	86.37		0.12	0.00-	0.25
	BRCA2	29	0	-						
Mutation type	None	142	1	Ref.			0.29	0.01	0.00-	0.04
	1991del4	7	0	-						
	3034del4	22	0	-						
	3450del4	54	3	13.12	1.36-	126.93		0.18	0.00-	0.34
	A1708E	26	0	-						

CI, confidence interval; Pval, P-value; Σrisk45y, cumulative risk by age 45 years; Ref., reference.

Supplementary Table 2. Estimated hazard ratios (HRs) of breast, ovarian and cervical cancer, and in relatives of carriers of Colombian *BRCA1/2* founder mutations stratified by birth year, proband's age at diagnosis, mutated *BRCA1/2* gene and *BRCA1/2* mutation type.

Variable	Level	Women	Events	HR	95% CI	Pval	Σrisk70y	95% CI
Birth year	Before 1960	65	11	Ref.		0.05	0.29	0.05- 0.47
	1960-69	59	14	4.03	1.53- 10.60		0.75	0.00- 0.95
	1970-79	53	1	2.62	0.27- 25.77		0.60	0.00- 0.95
	1980+	74	0	-				
Study type	Case-control	213	19	Ref.		0.31	0.33	0.11- 0.49
	Family study	38	7	1.57	0.66- 3.75		0.46	0.08- 0.69
Relationship with proband	Other	97	7	1.35	0.54- 3.40	0.05	0.47	0.02- 0.71
	Sister	86	13	Ref.			0.37	0.06- 0.58
	Daughter	57	5	3.71	1.28- 10.77		0.82	0.00- 0.98
	Mother	11	1	0.29	0.04- 2.25		0.13	0.00- 0.33
Proband's age at diagnosis	Less than 40	59	5	Ref.		0.62	0.32	0.00- 0.55
	40-44	57	6	1.16	0.35- 3.80		0.29	0.02- 0.48
	45-49	70	7	1.72	0.57- 5.14		0.44	0.02- 0.68
	50+	65	8	1.87	0.64- 5.42		0.47	0.04- 0.70
BRCA1/2 mutations	None	142	8	Ref.		0.0001	0.18	0.03- 0.31
	BRCA1	80	15	6.47	2.72- 15.41		0.73	0.30- 0.90
	BRCA2	29	3	2.41	0.64- 9.09		0.39	0.00- 0.66
Mutation type	None	142	8	Ref.		0.0003	0.19	0.03- 0.32
	1991del4	7	2	9.27	1.95- 44.00		0.86	0.00- 0.99
	3034del4	22	1	0.97	0.12- 7.76		0.18	0.00- 0.46
	3450del4	54	11	6.59	2.62- 16.56		0.75	0.26- 0.91
	A1708E	26	4	6.13	1.82- 20.60		0.72	0.00- 0.94

CI, confidence interval; Pval, P-value; Σrisk70y, cumulative risk by age 70 years; Ref., reference.

Supplementary Figure 1. Multiplex ligation-dependent probe amplification (MLPA) analysis of the *BRCA2* gene. (A), (B) Upper histograms: x-axis shows the lengths of the fragments (nt); ordinate shows the relative peak area (RPA) of the control result files as blue bars and the RPA of the patient result file as green bars. Lower histograms: x-axis shows the *BRCA2* exon probes and two *BRCA2* flanking probes (FRY, N4BP2L1 (CG018)); ordinate shows the RPA ratios (patient RPA divided by control RPA) as blue bars. (A) MLPA analysis of a normal control. The RPA ratios are in the range of 75-125% indicating the absence of a LGR. (B) MLPA analysis of a breast cancer patient. The RPA ratios are below 75% (0.40-0.59 and 0.41-0.72) indicating the presence of a heterozygous deletion of exons 1-14. Red arrows mark the deleted exons.

A

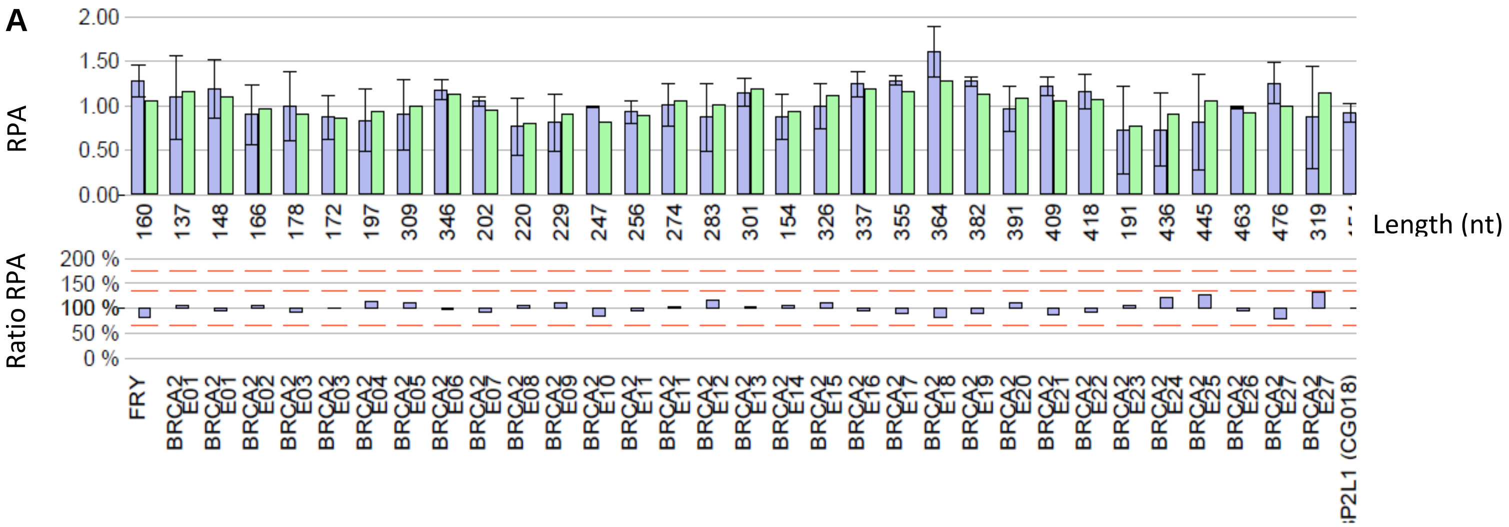

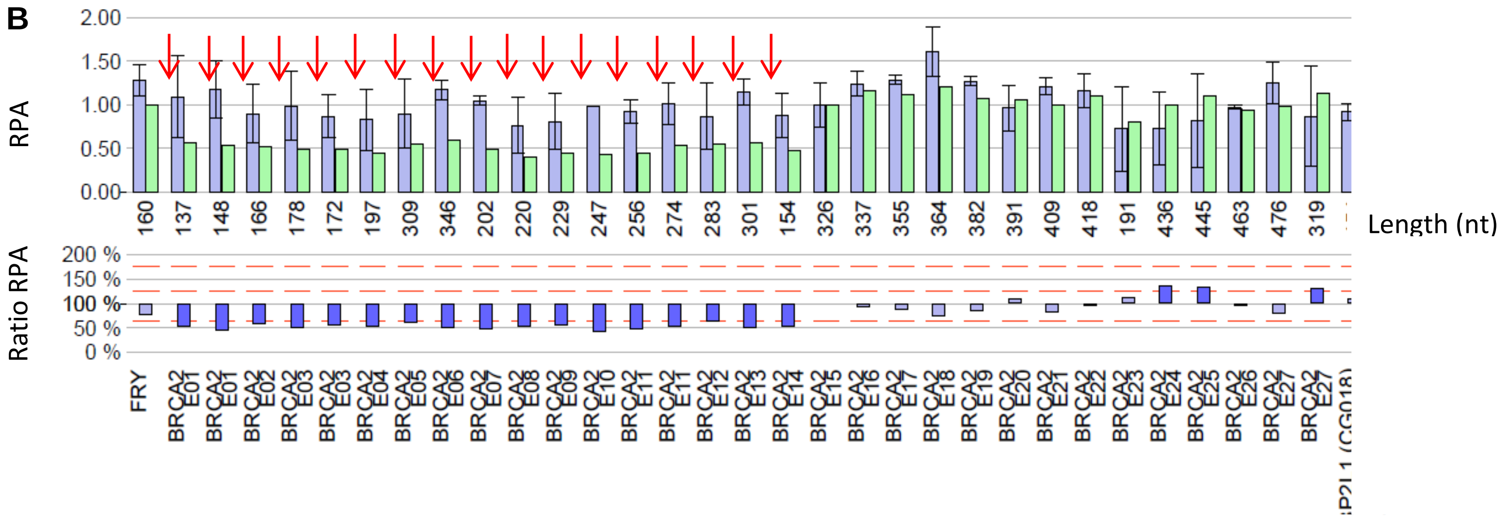
B
RPA
2.00
1.50
1.00
0.50
0.00
160 137 148 166 178 172 197 309 346 202 220 229 247 256 274 283 301 154 326 337 355 364 382 391 409 418 191 436 445 463 476 319
Length (nt)
Ratio RPA
200 %
150 %
100 %
50 %
0 %
FRY
BRCA2 E01
BRCA2 E01
BRCA2 E02
BRCA2 E03
BRCA2 E03
BRCA2 E04
BRCA2 E05
BRCA2 E06
BRCA2 E07
BRCA2 E08
BRCA2 E09
BRCA2 E10
BRCA2 E11
BRCA2 E11
BRCA2 E12
BRCA2 E13
BRCA2 E14
BRCA2 E15
BRCA2 E16
BRCA2 E17
BRCA2 E18
BRCA2 E19
BRCA2 E20
BRCA2 E21
BRCA2 E22
BRCA2 E23
BRCA2 E24
BRCA2 E25
BRCA2 E26
BRCA2 E27
BRCA2 E27

Supplementary Figure 2. Pedigree deviances revealed four departing pedigrees: two of them in favor of the alternative (with two carriers each diagnosed at ages 32 and 41, or 29 and 38), and two of them in favor of the null hypotheses (with a non-carrier diagnosed at age 39 and with one unaffected carrier at age 60). Exclusion of the four outlying families increased the HR of breast cancer by age 70 years.

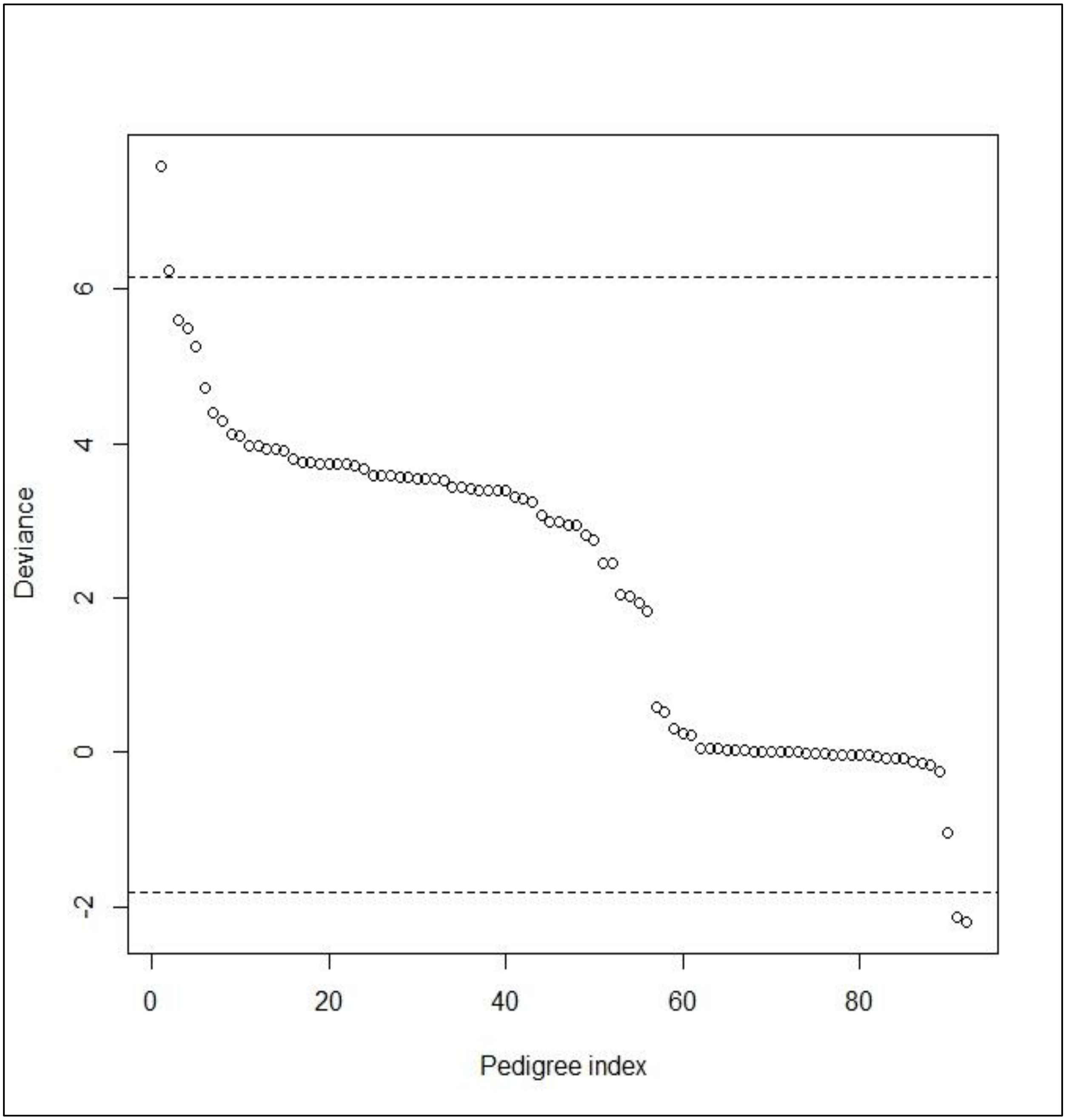

Supplement: Supplementary file 1 — Supplementary Material [file 41598_2017_5056_MOESM1_ESM.pdf]
